# Elucidating biogeographical patterns in Australian native canids using genome wide SNPs

**DOI:** 10.1371/journal.pone.0198754

**Published:** 2018-06-11

**Authors:** Kylie M. Cairns, Laura M. Shannon, Janice Koler-Matznick, J. William O. Ballard, Adam R. Boyko

**Affiliations:** 1 School of Biotechnology and Biomolecular Sciences, University of New South Wales, Sydney, New South Wales, Australia; 2 Department of Biomedical Sciences, Cornell University, Ithaca, New York, United States of America; 3 The New Guinea Singing Dog Conservation Society, Central Point, Oregon, United States of America; University of Southern Queensland, AUSTRALIA

## Abstract

Dingoes play a strong role in Australia’s ecological framework as the apex predator but are under threat from hybridization and agricultural control programs. Government legislation lists the conservation of the dingo as an important aim, yet little is known about the biogeography of this enigmatic canine, making conservation difficult. Mitochondrial and Y chromosome DNA studies show evidence of population structure within the dingo. Here, we present the data from Illumina HD canine chip genotyping for 23 dingoes from five regional populations, and five New Guinea Singing Dogs to further explore patterns of biogeography using genome-wide data. Whole genome single nucleotide polymorphism (SNP) data supported the presence of three distinct dingo populations (or ESUs) subject to geographical subdivision: southeastern (SE), Fraser Island (FI) and northwestern (NW). These ESUs should be managed discretely. The FI dingoes are a known reservoir of pure, genetically distinct dingoes. Elevated inbreeding coefficients identified here suggest this population may be genetically compromised and in need of rescue; current lethal management strategies that do not consider genetic information should be suspended until further data can be gathered. D statistics identify evidence of historical admixture or ancestry sharing between southeastern dingoes and South East Asian village dogs. Conservation efforts on mainland Australia should focus on the SE dingo population that is under pressure from domestic dog hybridization and high levels of lethal control. Further data concerning the genetic health, demographics and prevalence of hybridization in the SE and FI dingo populations is urgently needed to develop evidence based conservation and management strategies.

## Introduction

Dingoes are controversial in Australia; like many other top-order carnivores, dingoes pose a risk to livestock and are thus extensively managed in the livestock grazing regions of southeastern Australia [1]. There is also extensive debate concerning the taxonomy and definition of dingoes [2, 3]. However, dingoes are considered a native species, protected in national parks [4–7] and are listed as vulnerable on the IUCN Red List [8]. Conservation and management programs, adequately informed by scientific knowledge, must be developed to protect the identity of the dingo before it is lost.

As the mainland top-level predator, dingoes play a strong role in shaping the ecosystems of Australia [7, 9–11]. They have been observed to exert top-down control on large herbivores such as kangaroos, wallabies and emu [12–17] and may play a role in indirectly protecting native small-medium body weight marsupials [18–20]. In some cases, they may also reduce the impact of introduced feral mesopredator pests such as foxes and cats through suppression, exclusion and direct predation [17, 21–26].

**Fig 1:**
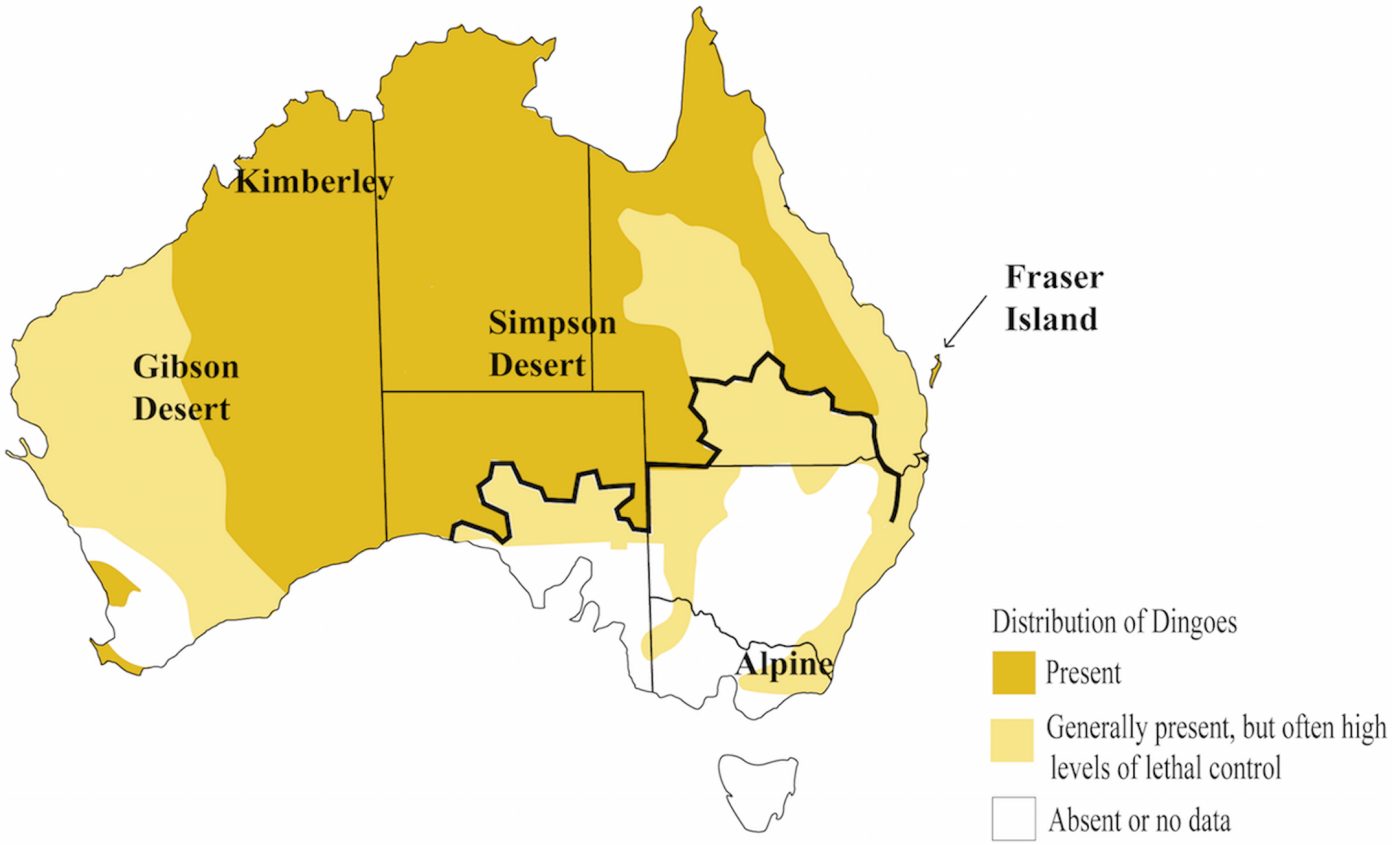
Distribution of dingoes across Australia. Map adapted from data in Fleming et al. [7] and Fleming et al. [36]. The bold black line indicates the position of the dingo fence; dingoes south of the dingo fence are particularly subject to high levels of lethal control and may have a higher prevalence of hybridization. Broad geographic sampling regions are noted on the map: the Kimberley, the Gibson Desert, the Simpson Desert, Fraser Island and the Australian Alpine region.

In many parts of Australia dingoes are subject to lethal control in an effort to mitigate risks to agricultural activities [7, 27, 28]. However, lethal control and management practices may not always decrease dingo population size and pack destabilization may result in increased levels of hybridization [16, 29]. Similarly, collapse of dingo social structures may increase livestock predation risk [16, 30]. Dingoes are widespread across the Australian mainland, although rare in some areas due to high level of lethal control (Fig 1) [7]. Genetic evidence has raised concern that dingoes are at risk of genetic dilution through hybridization with European domestic dogs [31–35]. Ultimately, management decisions regarding dingoes made without data regarding the genetic identity and health of a population may have widespread ecological implications.

Knowledge concerning the genetic health of natural populations, particularly isolated or threatened populations, is important for the development of effective conservation and management programs. Severe inbreeding is a particular risk to conservation efforts because it may lead to inbreeding depression, leaving a population more vulnerable to environmental or demographic fluctuations, and possibly resulting in extinction [37–43]. Genetic rescue may be used to improve the fitness of threatened species or populations [44]. Inbreeding is of particular concern for the Fraser Island dingo population given their low effective population size, conservation significance and the lethal management strategies employed [32, 45–47].

Current genetic phylogenies based upon mitochondrial, Y chromosome and whole genome single nucleotide polymorphism (SNP) data suggest that dogs, wolves and dingoes form a monophyletic group [48–54]. Dogs likely diverged from wolves 16,000–30,000 years before present (BP) [51, 55–61]. Dingoes diverged from other dogs approximately 5,000–10,000 years BP [50, 52, 53, 62] and arrived in Australia at least 5,000 years BP [63–66]. Genetic comparisons between dingoes and other canids suggest that dingoes have an affinity with Asian wolves and dogs, hinting that their heritage is most likely Asian [54]. It is hypothesized that dingoes migrated into Australia through South East Asia [48, 50, 52, 62, 67].

Previous genetic studies have focused largely upon uniparental haplotypes, using mitochondrial and Y chromosome markers. Mitochondrial control region studies asserted that modern dingo populations were likely the result of a single very homogeneous founder population, possibly even a single pregnant female [53]. However, Sacks et al. [52], Ardalan et al. [48] and Cairns et al. [67] detected the presence of two divergent paternal genetic lineages within dingoes. Further, Cairns and Wilton [62] observed the presence of two geographically subdivided populations of dingo using mitochondrial markers and a small number of autosomal loci. However, uniparental markers may be maternally or paternally biased and show different evolutionary patterns to autosomal markers [68–72]. Here we focus on patterns of genetic diversity in 23 dingoes sampled from five geographical populations across Australia and using nearly 60,000 SNPs to improve our understanding of population structure and genetic diversity in this unique canid. Specifically, we aim to describe patterns of genetic subdivision in dingoes using SNP data. We also investigate patterns of ancestral sharing with domestic dogs to develop hypotheses concerning the origins and modern history of dingoes, particularly about the prevalence of hybridization with European dogs. Inbreeding data are also interrogated to inform hypotheses concerning the genetic health of dingo populations on the Australian mainland and on Fraser Island. We discuss the implications of this data for the ongoing management and conservation of dingoes.

## Materials and methods

All applicable international, national, and/or institutional guidelines for the care and use of animals were followed. This research was approved by the Animal Care and Ethics Committee of the University of NSW (Permit Number: 12/36B).

### Canid sampling

To investigate patterns of biogeography, we sampled 25 wild dingoes from five geographical regions: The Kimberley (Western Australia), The Gibson Desert (Western Australia), The Simpson Desert (Northern Territory), Fraser Island (Queensland) and the Australian Alpine region (Australian Capital Territory, Victoria and New South Wales) (S1 Table; Fig 1). Dingoes were sampled from these five geographical regions to capture genetic variation across the continent; these are the same regions sampled by Cairns and Wilton [62] and Cairns et al. [67]. DNA was extracted from blood or tissue samples using the Qiagen DNeasy Blood and Tissue kit according to the manufacturer’s instructions (Qiagen Sciences, Germantown, USA). All dingo samples were verified as genetically ‘pure’ using a 23-marker DNA test developed for distinguishing dingoes from dingo-dog hybrids [33, 34].

### Illumina HD canine genotyping

Samples were genotyped on the 170K Illumina HD Canine SNP array (Illumina Inc., San Diego, USA) at the Cornell Genomics Core Facility (Cornell University, Ithaca, USA). Genotypes were called using GenomeStudio (Illumina Inc., San Diego, USA) and quality control filtering was conducted in PLINK v1.7 [73]. Specifically, individuals missing more than 10% of SNPs were excluded, SNP sites with more than 10% missing data were excluded and SNPs with a minor allele frequency of less than 5% were excluded (resulting in 23 remaining samples). For some genetic analyses (Table 1), previously published genotype data from 5 New Guinea singing dogs and/or 12 wolves (*Canis lupus*) were included [57, 74]. To investigate the effect of domestic dog introgression further analyses included a set of 35 domestic dog samples: 8 Australian cattle dogs, 8 Borneo village dogs, 9 Vietnam village dogs and 10 Portugal village dogs [57]. Australian cattle dogs were specifically incorporated as representative of modern Australian domestic dog breeds. When combining datasets, genotype data were merged un-filtered and then filtering steps were completed, as above.

**Table 1:** Description of datasets and samples used in analyses.

Dataset	Samples	Analyses
‘Dataset A’	23 Dingoes + 5 NGSD	Inbreeding, Clustering, PCA and Phylogenetics
‘Dataset B’	23 Dingoes + 5 NGSD + 35 Dogs	Clustering and PCA
‘Dataset C’	23 Dingoes + 5 NGSD + 12 Wolves	Phylogenetics
‘Dataset D’	23 Dingoes + 12 Wolves + 35 Dogs	Introgression modelling

### Inbreeding and homozygosity

Using ‘Dataset A’, a sex check analysis was also performed in PLINK to confirm gender assignments. As dingoes and NGSD are closely related [59], inbreeding statistics were calculated using ‘Dataset A’. As such, individual inbreeding coefficients (equivalent to Wright’s *F*_*IS*_), examining differences in the observed and expected homozygosity levels for each individual were calculated in PLINK v1.7 [73]. F_IS_ is a relative comparison between a sample and the reference populations expected and observed homozygosity. Individual inbreeding coefficients were then averaged across geographical populations. Individual inbreeding coefficients were also calculated for only dingoes versus only NGSDs, although it is important to note that NGSDs are known to be inbred based on pedigree.

### Clustering analysis

ADMIXTURE v1.23 [75] was used to perform maximum likelihood clustering analyses based on autosomal markers. Clustering analyses were run with the following conditions: 10-fold cross-validation and iterations for each K were run until the change in the log likelihood value was below 0.1. Ten independent runs of each K value were completed, each using different random seeds. The best K value was chosen by comparing the cross validation errors for each K value between the independent runs [75]. CLUMPP v1.1.2 was used to compare and average the Q matrices of the ten independent runs for this K value [76]. An average Q-plot for the best K(s) was constructed using Distruct v1.1 [77]. The resulting Q-plot is a consensus of the possible scenarios (or modes) for a specific K value. First, analyses were run using ‘Dataset A’. A map depicting the population assignment of each sample was created using the maps package [78] in R v3.2.1 [79]. *F*_*ST*_ values between the four population clusters were calculated in ADMIXTURE v1.23 [75]. Clustering analyses were then repeated, as above, using ‘Dataset B’, to investigate the possibility of introgression from domestic dogs into dingoes.

### Principal components analysis

A principal components analysis (PCA) was performed on ‘Dataset A’ in PLINK v1.9 [73, 80]. The top 20 eigenvalues and eigenvectors were calculated. The percentage variation that each principal component (PC) vector accounts for was calculated using the following formula: eigenvalue/(Σ of all eigenvalues) × 100. The top three PCA eigenvectors, accounting for the largest percentage variance, were plotted using the rgl package [81] in R v3.2.1 [79]. To answer questions concerning the likelihood of domestic dog introgression in dingoes the PC analyses were repeated using ‘Dataset B’.

### Phylogenetic analyses

The phylogenetic relationships between dingoes and NGSDs were investigated using SNPhylo [82] a pipeline for constructing maximum likelihood (ML) trees from genome wide SNP datasets. First, ‘Dataset A’ was pruned for invariant SNPs and the remaining ancestry informative SNPs were concatenated together in SNPhylo [82]. This pruned data was used for phylogenetic analyses. Allele frequencies and association statistics were calculated in PLINK for ancestry informative markers, as identified by SNPhylo [82] to investigate their utility in future genetic studies. SNPs with Wald test values of *p* <10^−4^ were considered to be strongly associated with population structure in the dingo and NGSD.

Concatenation is a method of combining sequences from multiple genetic loci and is particularly useful for intraspecific datasets where divergences may be recent [83]. However concatenation may introduce biases as a result of rate heterogeneity, differences in gene tree topology and/or recombination [84]. Filtering of invariant sites, as employed by SNPhylo, may also bias branch lengths. As such phylogenies should be treated conservatively. As implemented by the SNPhylo pipeline [82], an unrooted ML tree using a Hidden Markov Model was constructed in DNAml [85]. Non-parametric bootstrapping with 6000 repetitions was performed on the ML tree using Phangorn [86] as implemented in the SNPhylo pipeline [82].

Additionally, SNPhylo [82] was used to create a pruned and ancestry informative concatenated sequence for each sample in ‘Dataset C’. A rooted ML analysis was then run in raxmlGUI [87] with a GTR + G substitution model and 2000 bootstrap replicates.

### Introgression modeling

To investigate the extent of modern domestic dog introgression into dingoes D-statistics were calculated using ADMIXTOOLS [88]. This analysis was run using Dataset D. The wolf population (the *W* population) incorporated samples from four geographical wolf populations: European, Chinese, Middle Eastern, and Russian. Data from dog populations (the *X* populations) in Borneo, Vietnam, Portugal and from the Australian cattle dog breed were included in the analysis [57]. In the analysis, Alpine dingoes were the *Y* population and the non-alpine dingo populations were the *Z* population. Standard error and Z-statistics were also calculated.

## Results

### Illumina HD canine genotyping

Of the 25 dingoes genotyped, two were excluded from the analyses due to >10% missingness. The remaining 23 dingoes from 5 geographical populations and 5 NGSDs (Table 2, doi:10.5061/dryad.sq8d0) had a genotyping rate before filtering of 0.974, and 0.990 after filtering. A total of 58,512 autosomal SNPs remained after filtering. A sex check performed in PLINK confirmed the gender identity of samples.

**Table 2:** Gender and individual inbreeding coefficients for 23^a^ dingoes and 5 NGSD (‘Dataset A’).

ID	Geographical population	Sex	*F*_*IS*_ *(only dingoes or only NGSD)*	*F*_*IS*_ *(dingoes and NGSD)*	Average *F*_*IS*_ *(dingoes and NGSD)*
Alpine 1	Alpine	M	-0.033	0.002	0.089
Alpine 2	Alpine	M	-0.011	0.038	
Alpine 3	Alpine	M	0.096	0.137	
Alpine 4	Alpine	F	0.297	0.331	
Alpine 5	Alpine	M	-0.1	-0.060	
Fraser 3	Fraser	M	0.715	0.732	0.700
Fraser 4	Fraser	M	0.625	0.647	
Fraser 5	Fraser	F	0.704	0.720	
Fraser 7	Fraser	M	0.683	0.702	
Gibson 1	Gibson	F	0.406	0.439	0.240
Gibson 2	Gibson	F	0.072	0.122	
Gibson 3	Gibson	M	0.053	0.100	
Gibson 4	Gibson	F	0.208	0.250	
Gibson 5	Gibson	F	0.144	0.188	
Northwestern 2	Gibson	M	0.305	0.339	
Kimberley 1	Kimberley	F	0.234	0.271	0.215
Kimberley 2	Kimberley	M	0.18	0.218	
Kimberley 3	Kimberley	M	0.103	0.142	
Kimberley 4	Kimberley	F	0.237	0.270	
Northwestern 9	Kimberley	F	0.138	0.175	
Simpson 1	Simpson	F	0.122	0.161	0.138
Simpson 2	Simpson	M	0.053	0.097	
Simpson 5	Simpson	F	0.119	0.157	
NGSD A	NGSD	M	-0.289	0.457	0.561
NGSD B	NGSD	M	0.218	0.671	
NGSD C	NGSD	M	0.034	0.594	
NGSD D	NGSD	F	0.096	0.620	
NGSD E	NGSD	M	-0.28	0.461	

^a^ two dingoes of the original 25 were excluded for failing to adequately run (Fraser 6) or having more than 10% missing SNPs (Simpson 3).

### Inbreeding and homozygosity

**Fig 2:**
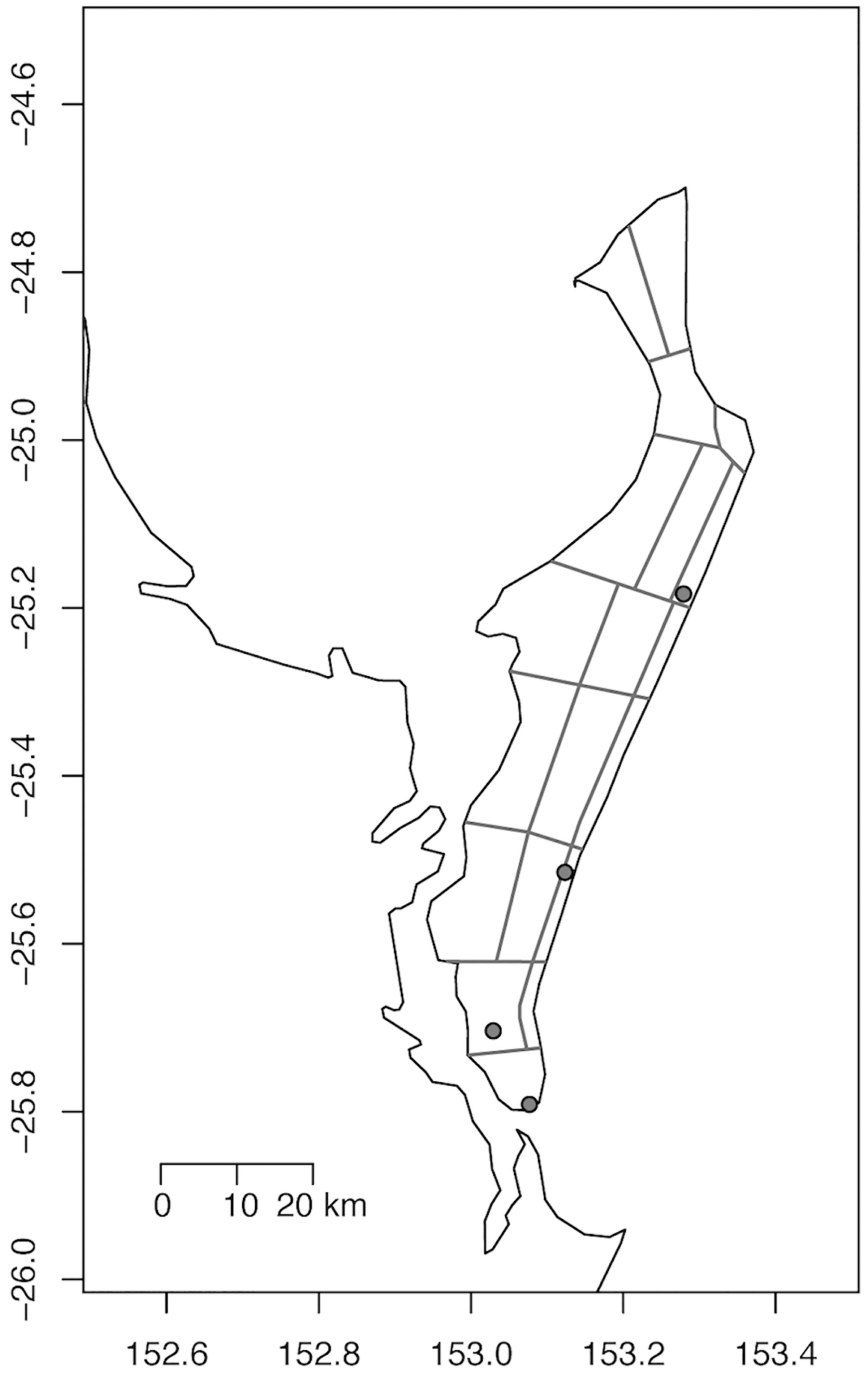
Distribution of Fraser Island dingo samples and natal pack territories. The location of dingo samples is indicated by enclosed grey circles and the boundaries of estimated natal pack territories adapted from Allen et al. [89] are drawn in dark grey.

Individual inbreeding coefficients (*F*_*IS*_) calculated based on 58,512 autosomal SNP loci suggested that some dingo populations were inbred (Table 2). The four Fraser Island dingoes had very high *F*_*IS*_ values of 0.647–0.732 indicating an extreme level of inbreeding. Sampling locations for Fraser Island samples indicate that the dingoes came from different natal pack territories (Fig 2). Similarly, the NGSD population was highly inbred with *F*_*IS*_ values ranging from 0.457–0.671. The Alpine and Simpson Desert populations had the lowest average *F*_*IS*_ statistics. When inbreeding coefficients were calculated using only dingo data the values only changed mildly. However, inbreeding coefficient values calculated using only NGSD data were different from those calculated with dingo data; this is likely because all sampled NGSD were inbred (as indicated by pedigree).

### Clustering analyses

**Fig 3:**
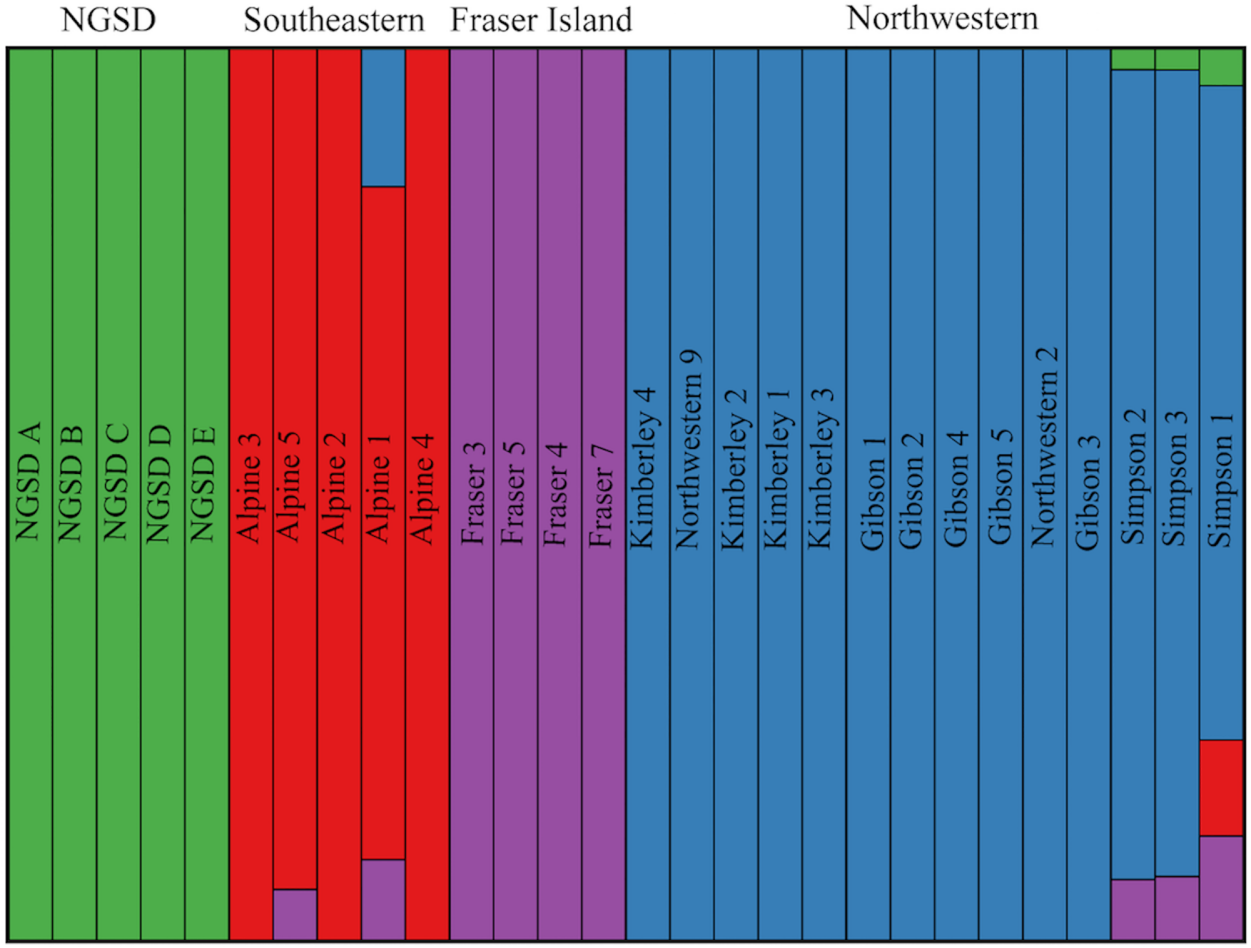
Maximum likelihood population clustering analysis on ‘Dataset A’ (23 dingoes and 5 NGSD) at 58,512 SNP loci. Average Q-plot for K = 4 constructed in Distruct v1.1 [77]. Each column represents an individual and the proportion population cluster identity. Population clusters are represented by colours: green for New Guinea Singing Dog, red for southeastern, purple for Fraser Island and blue for northwestern.

**Fig 4:**
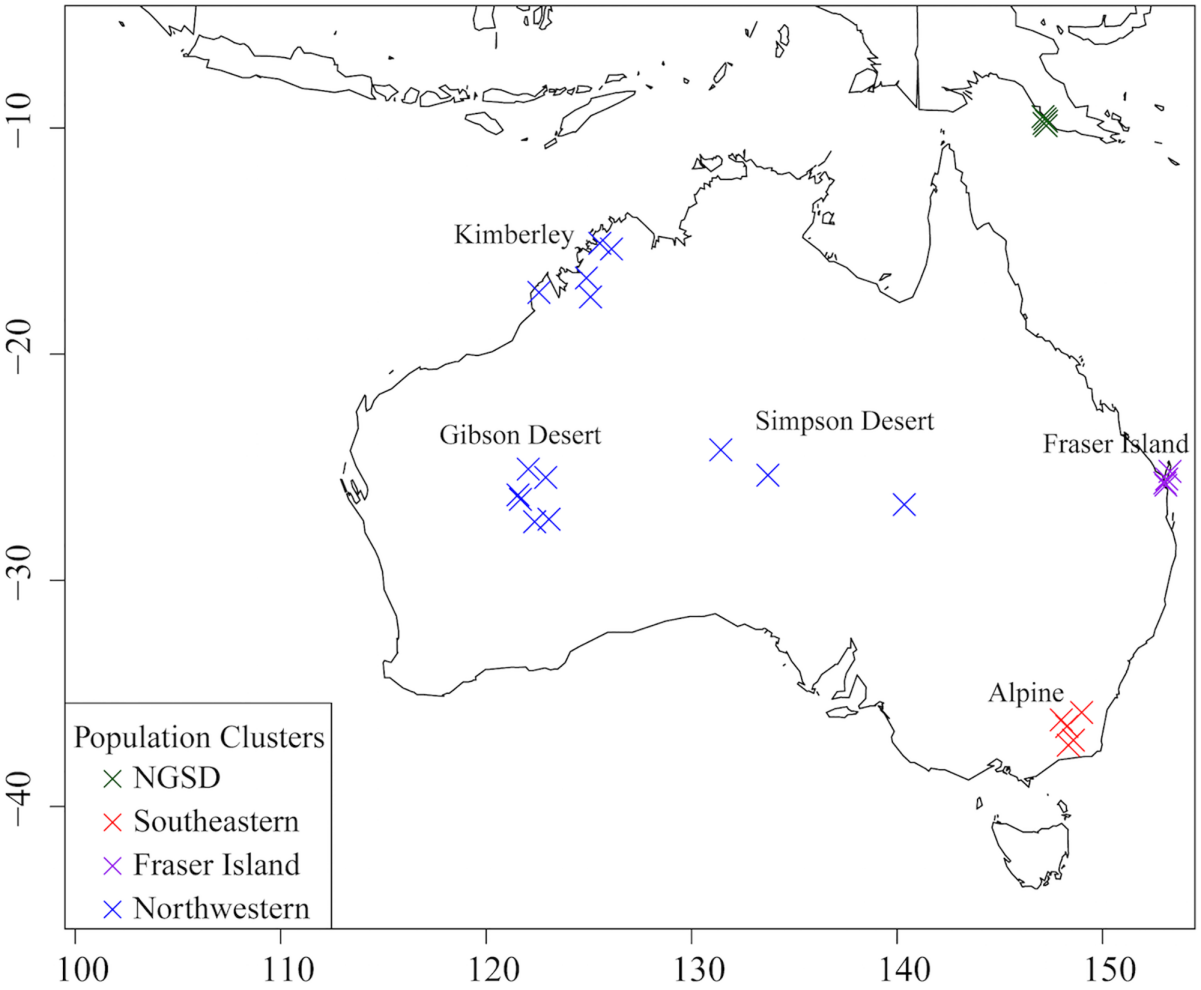
Geographical map depicting sampling location of each sample from ‘Dataset A’ and its majority population cluster identity. NGSD samples (plotted in Papua New Guinea) are from a captive North American population.

For ‘Dataset A’, clustering analyses indicated the best K was 4 (S1 Fig). ADMIXTURE analyses suggest the presence of four population clusters in ‘Dataset A’: southeastern (SE), northwestern (NW), Fraser Island (FI) and New Guinea Singing Dog (NGSD) (Figs 3 and 4). There is some evidence of possible ancestry sharing between population clusters in some individuals, particularly Alpine 1, Alpine 5 and the three Simpson Desert dingoes. *F*_*ST*_ values between the four population clusters indicate a high level of differentiation and low gene flow between the populations (Table 3).

**Table 3:** *F*_*ST*_ values between dingo and NGSD populations (‘Dataset A’). Calculated by ADMIXTURE (v1.23) based on 58,512 SNP loci.

	**NGSD**	**Fraser Island**	**Southeastern**
**NGSD**	-	-	-
**Fraser Island**	0.408	-	-
**Southeastern**	0.354	0.61	-
**Northwestern**	0.238	0.431	0.421

**Fig 5:**
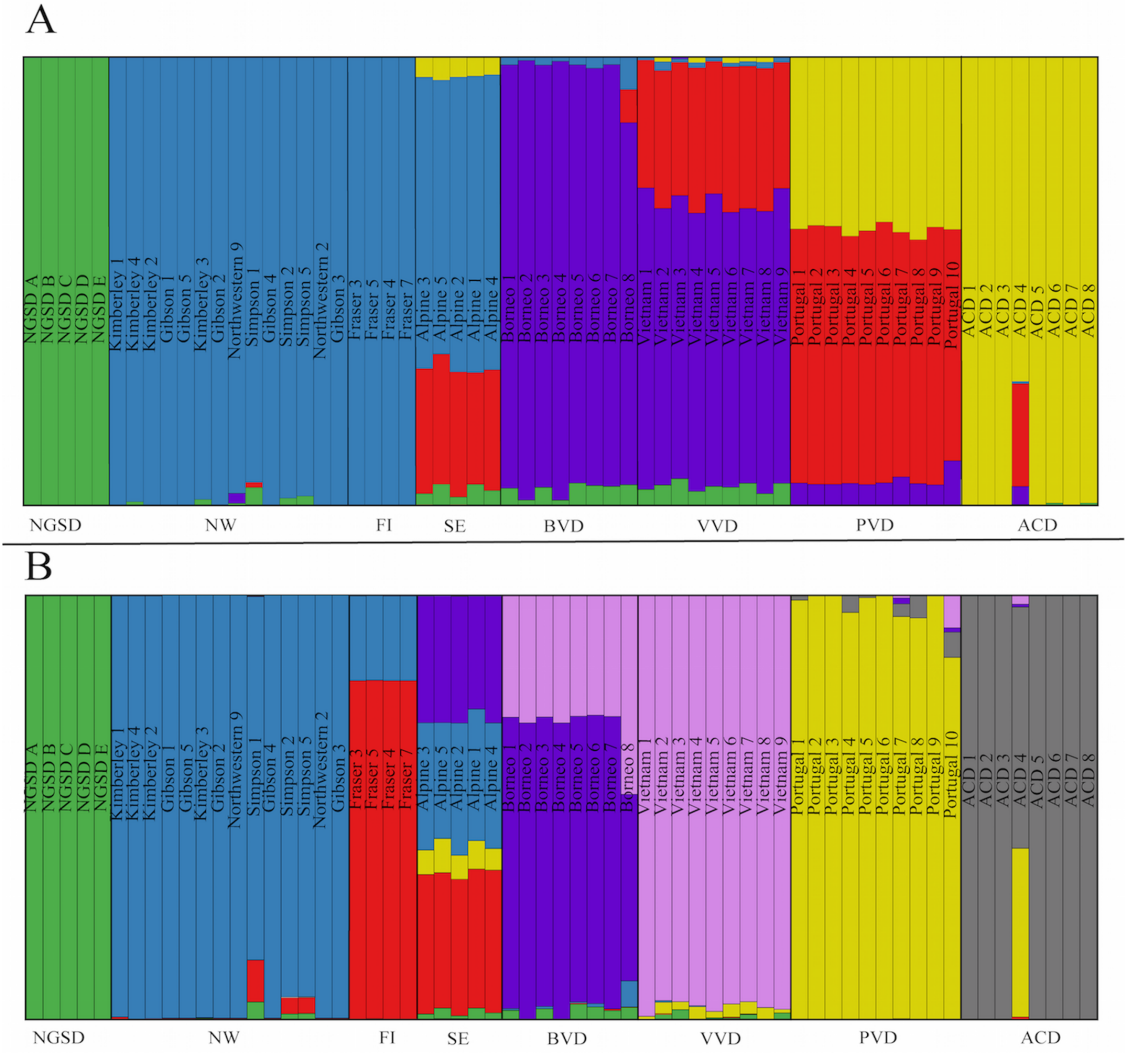
Maximum likelihood population clustering analysis on 23 dingoes, 5 NGSD, 8 Borneo village dogs, 9 Vietnam village dogs, 10 Portugal village dogs and 8 Australian cattle dogs (‘Dataset B’) at 58,512 SNP loci. Average Q-plots constructed in Distruct v1.1 [77]. Each column represents an individual and the proportion population cluster identity. Abbreviations represent populations: NGSD for New Guinea Singing Dog; NW for northwestern dingoes; FI for Fraser Island dingoes; and SE for southeastern (Alpine) dingoes; BVD for Borneo village dogs; VVD for Vietnam village dogs; PVD for Portugal village dogs and ACD for Australian cattle dogs. **(A)** Average Q-plot for K = 5. **(B)** Average Q-plot for K = 7.

For ‘Dataset B’, analyses indicated the best K was 5 (S2 Fig). Results for K = 7 are also presented because this represents the number of geographical populations. Clustering analyses incorporating a set of domestic and village dogs are inconsistent between values of K (Fig 5). Modeling for K = 5 and K = 7 present conflicting data for Alpine dingoes. In K = 5 Alpine dingoes share some ancestry with Vietnam village dogs and Portugal village dogs (Fig 5). However, when K = 7, we see significant ancestry sharing between the SE dingoes and Borneo village dogs (Fig 5).

### Principal components analysis

**Fig 6:**
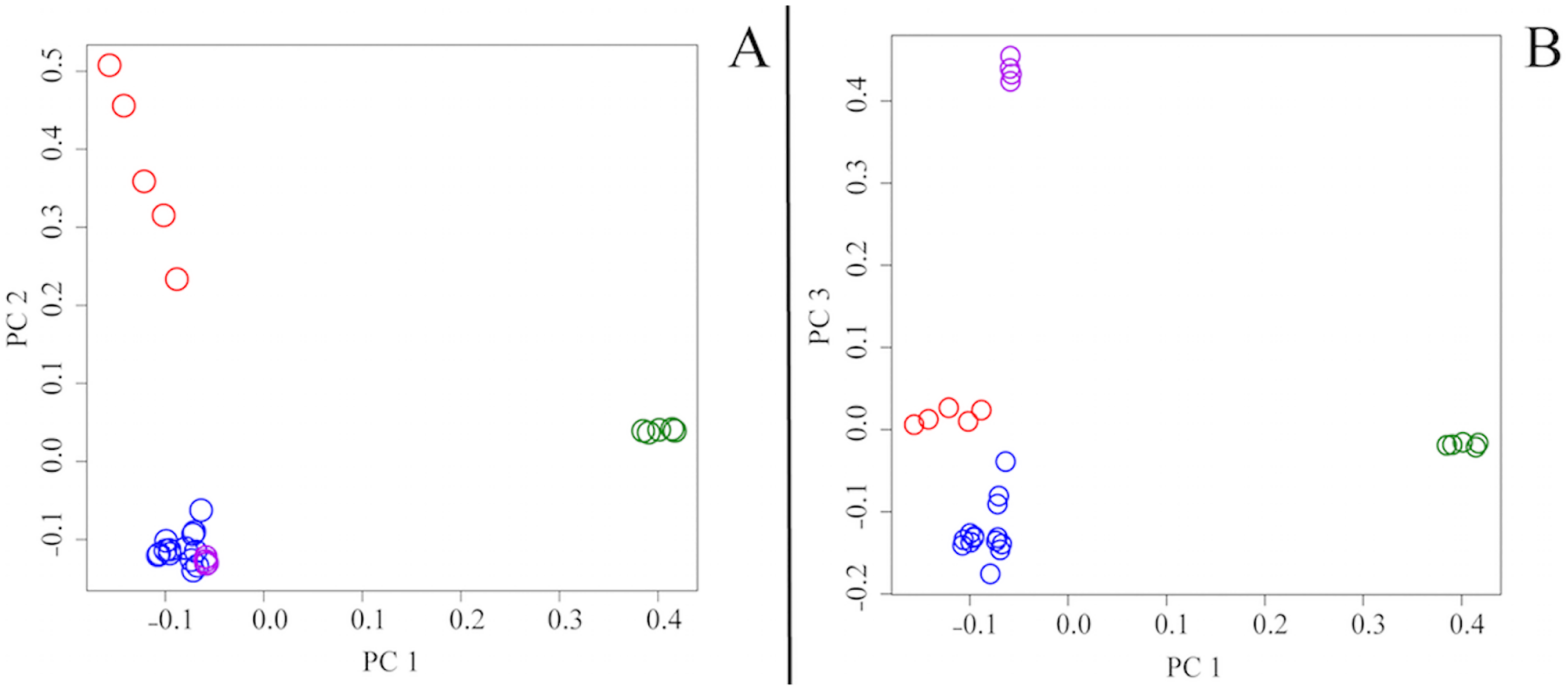
Principal components analysis (PCA) based upon filtered whole genome SNP genotypes (58,512 sites) for 23 dingoes and 5 NGSD. Colours represent population clusters: red for SE dingoes, purple for FI dingoes, blue for NW dingoes and green for NGSD. **(A)** PC 1 versus PC 2. (**B)** PC 1 versus PC 3.

For ‘Dataset A’, the top three PC vectors account for 20.5% (eigenvalue = 6.872), 15.1% (eigenvalue = 5.060) and 9.8% (eigenvalue = 3.276) of the genetic variance respectively and indicate the presence of four population clusters: SE, FI, NW and NGSD. PC1 differentiates NGSDs from dingoes, PC2 separates out SE dingoes and PC3 distinguishes FI dingoes from the other populations (Fig 6). The three eastern most NW dingoes (from the Simpson Desert) cluster slightly closer to the SE dingoes, a result of either historical mixing between the SE and NW populations in this region or isolation by distance (Figs 4 and 5).

**Fig 7:**
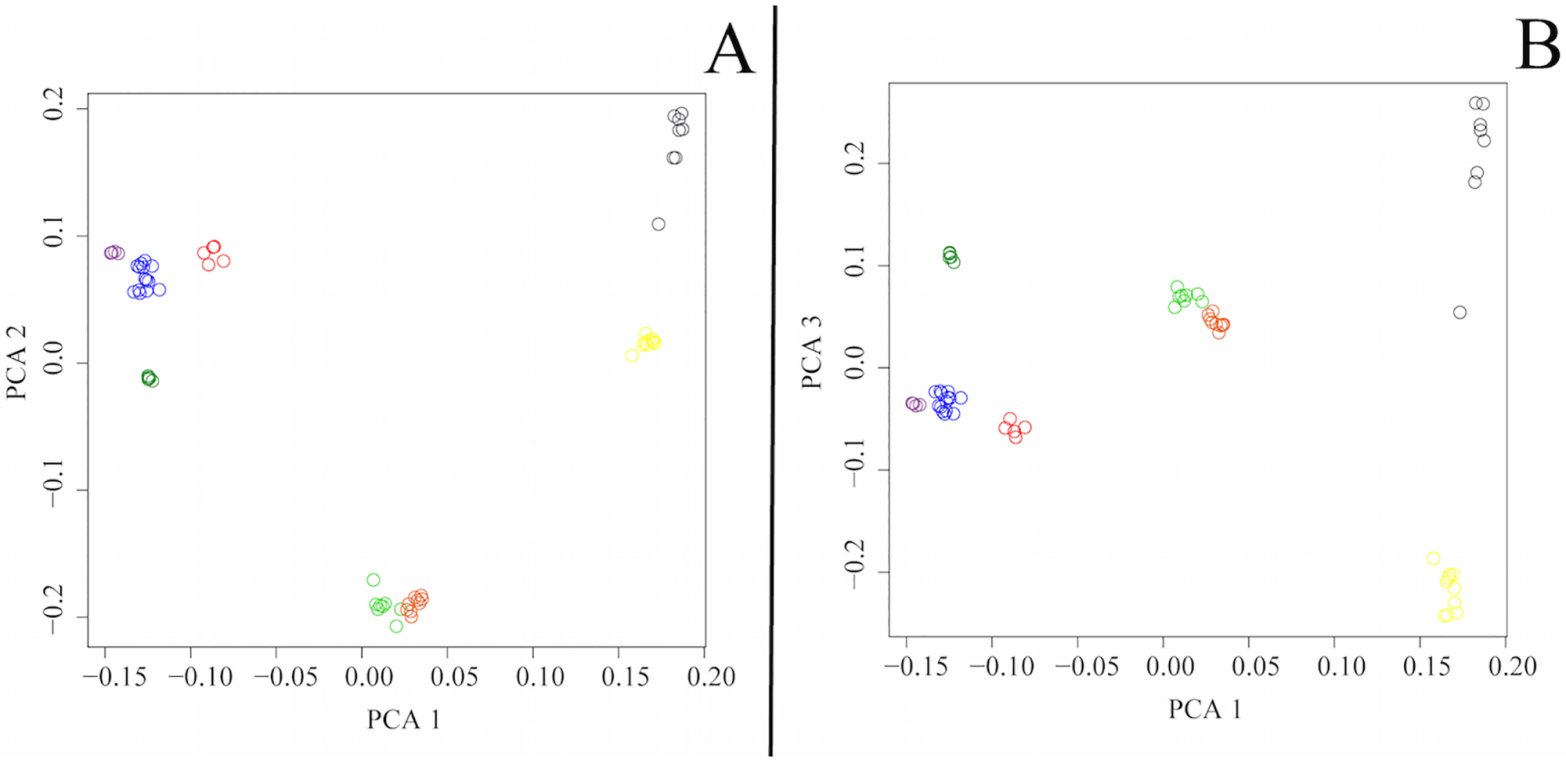
Principal components analysis (PCA) based upon filtered whole genome SNP genotypes (58,512 sites) for 23 dingoes, 5 NGSD, 8 Borneo village dogs, 9 Vietnam village dogs, 10 Portugal village dogs and 8 Australian cattle dogs (‘Dataset B’). Colours represent population clusters: red for SE dingoes, purple for FI dingoes, blue for NW dingoes, dark green for NGSD, light green for Borneo village dogs, orange for Vietnam village dogs, yellow for Portugal village dogs and grey for Australian cattle dogs. **(A)** PC 1 versus PC 2. (**B)** PC 1 versus PC 3.

When PC analyses were repeated with ‘Dataset B’, we see the same four population clusters: SE, FI, NW and NGSD as well as three new clusters representing the Vietnam and Borneo village dogs, Portuguese dogs and Australian cattle dogs. The top three PC vectors accounted for 37.8% (eigenvalue = 20.763), 9.1% (eigenvalue = 4.992) and 5.5% (eigenvalue = 3.044) of the genetic variance respectively. PC1 differentiates dingoes and NGSD from both Asian village dogs and European dogs while PC2 and PC3 distinguish NGSD from dingoes and Portuguese village dogs from ACD (Fig 7). It is interesting to note that NGSD and dingoes are well separated from European dogs.

### Phylogenetic analyses

**Fig 8:**
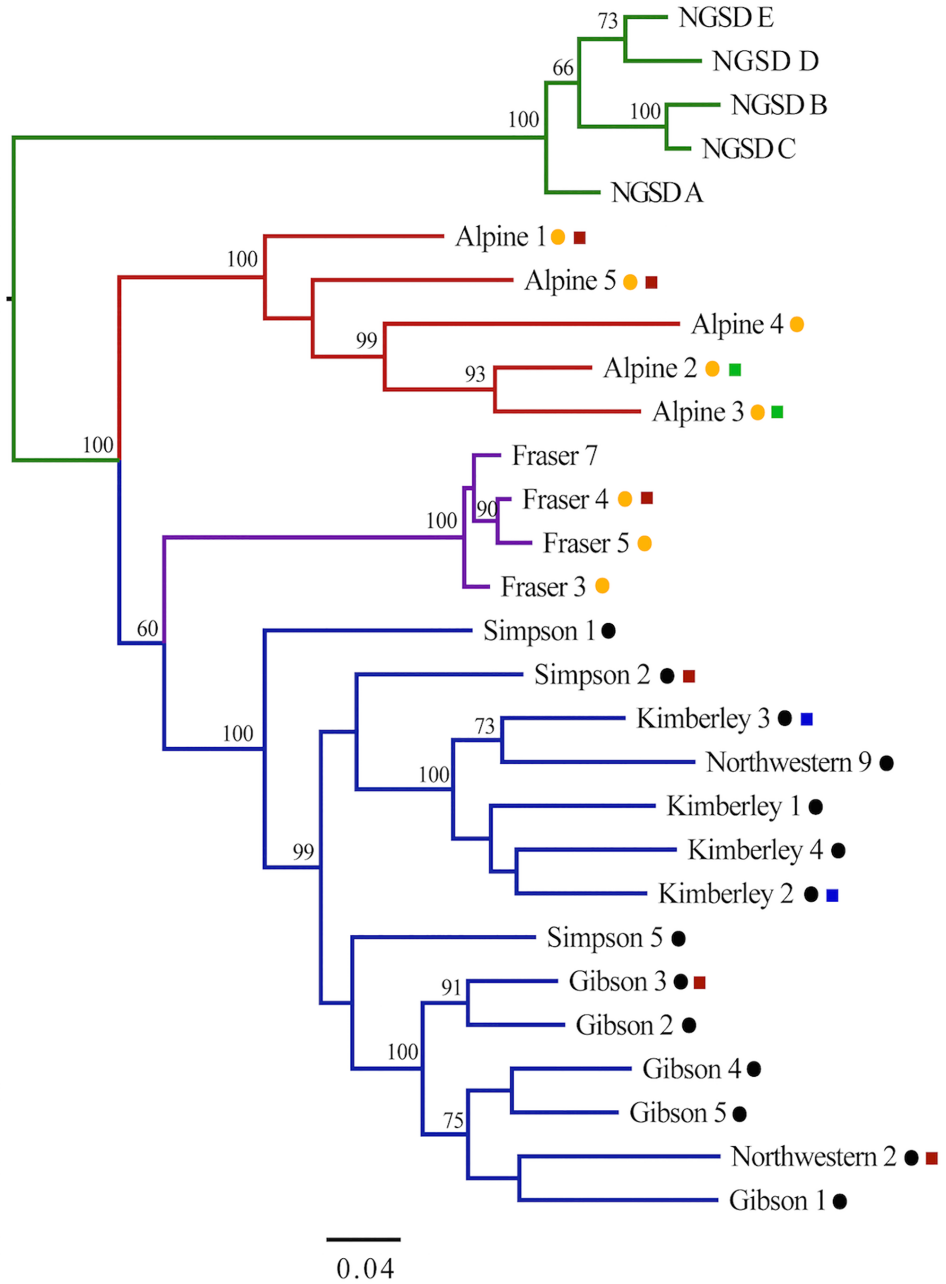
Maximum likelihood tree based upon 4,913 ancestry informative markers in 23 dingoes and 5 NGSD (‘Dataset A’). The tree was constructed via the SNPhylo pipeline [82], with 6,000 non-parametric bootstrap replicates. Bootstrap values located above nodes, values below 60 not shown. Colours represent population clusters: red for SE dingoes, purple for FI dingoes, blue for NW dingoes and green for NGSD. Circles indicate mitochondrial lineage with; black for NW and orange for SE [62]. Squares depict Y chromosome haplogroup with; green for H1, blue for H3 and red for H60 [67].

For ‘Dataset A’, SNPhylo identified 4913 SNPs that were variable and ancestry informative. Of these a total of 460 SNPs were identified has having significant Wald test values (*p*<10^−4^) indicating a strong association between these SNPs and geographical population. These SNPs may be useful for developing a SNP assay to investigate population structure in a larger geographic survey of dingoes. The unrooted ML tree constructed in DNAml [85] identified four major populations: SE dingoes, FI dingoes, NW dingoes and NGSD (Fig 8). However, bootstrap support for the split between FI and NW dingoes is 60 indicating phylogenetic uncertainty.

**Fig 9:**
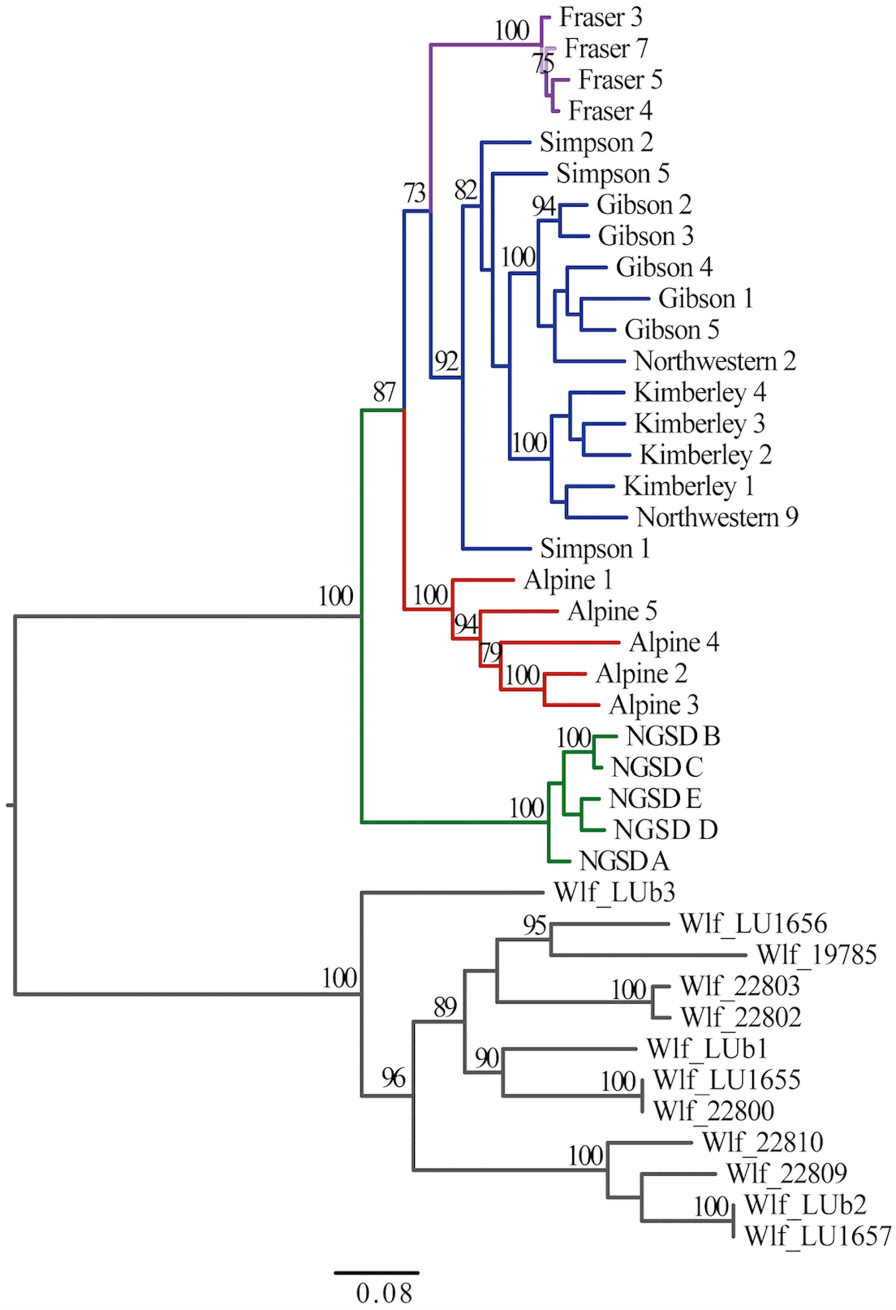
Maximum likelihood tree constructed based upon 6,288 informative SNPs in 23 dingoes, 5 NGSD and 12 wolves (‘Dataset C’). The 12 wolf samples [74] were added as outgroup taxa. Tree constructed in raxmlGUI [87] using a GTR + G substitution model and 2000 bootstrap replicates. Bootstrap values located above nodes, values below 70 not shown. Colours represent population clusters: red for SE dingoes, purple for FI dingoes, blue for NW dingoes, green for NGSD and gray for wolves.

To further explore the phylogenetic relationship between dingoes and NGSD a second rooted analysis was completed using ‘Dataset C’, this incorporated 12 wolf (*Canis lupus*) samples as outgroup taxa. The rooted ML analysis in raxmlGUI [87] confirmed that NGSD form their own monophyletic group compared to dingoes (Fig 9) suggesting that the NGSD diverged before the dingo populations differentiated. Additionally, the genome-wide SNP phylogeny indicates that the FI dingo population is closely related to the NW dingo populations with bootstrap support of 73 (60 in unrooted phylogeny), representing phylogenetic uncertainty. The split between the SE and NW dingo populations is strongly supported with a bootstrap value of 87 (100 in unrooted phylogeny).

### Introgression modeling

**Fig 10:**
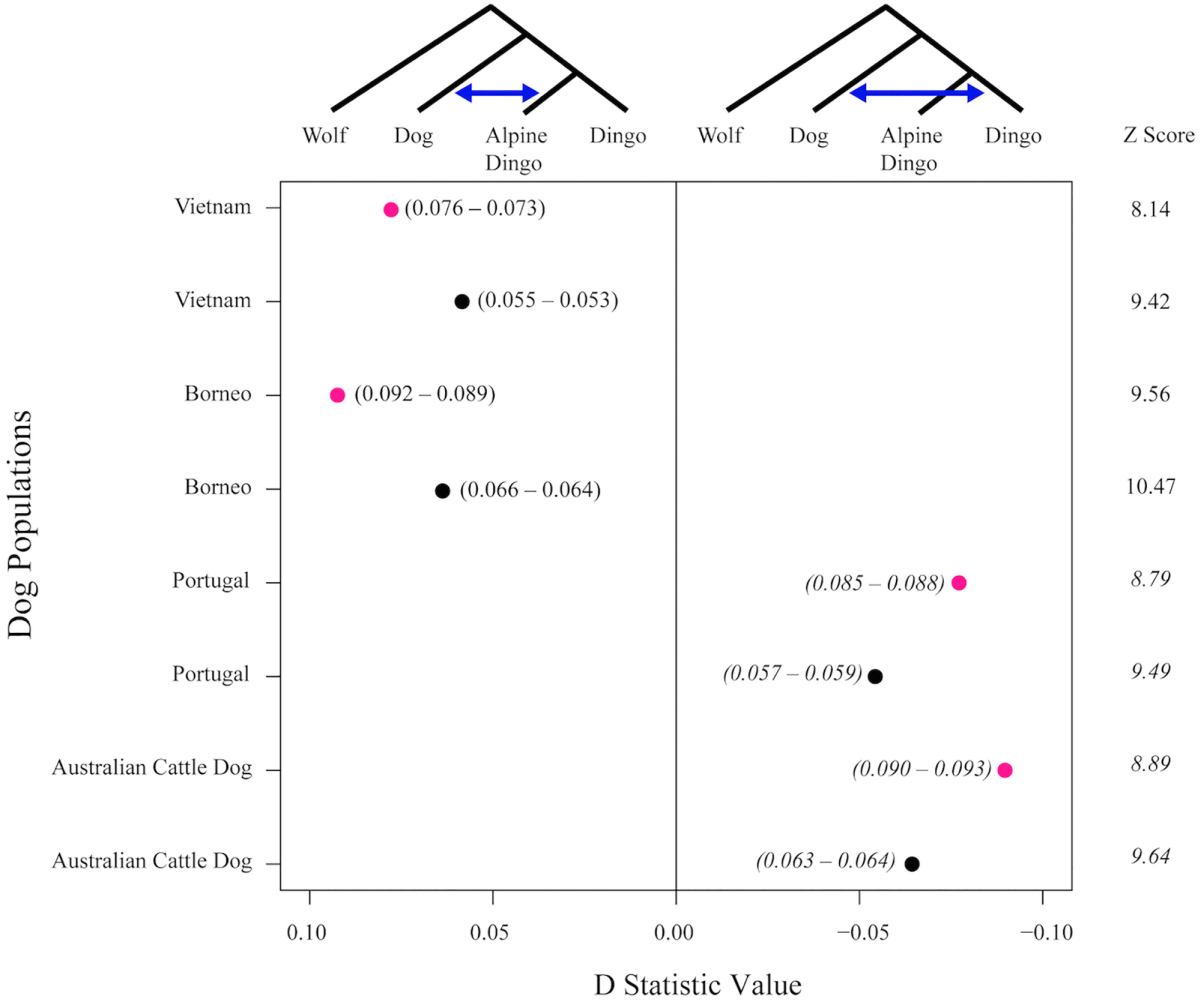
Introgression testing using D statistics based on 58,512 SNP sites for 23 dingoes, 5 NGSD, 8 Borneo village dogs, 9 Vietnam village dogs, 10 Portugal village dogs, 8 Australian cattle dogs and 12 wolves (‘Dataset D’). The topology tested was *W* (wolves), *X* (dogs), *Y* (Alpine dingoes) and *Z* (non-Alpine dingoes). The non-Alpine dingo populations (*Z* population) are represented as pink dots for Fraser Island dingoes and black dots for northwestern dingoes. Values next to dots indicate the range of D statistic values with error; italics indicate negative D statistic values.

Using ‘Dataset D’, D statistics were used to investigate the possibility of introgression from domestic dogs into dingoes. Standard error was low and Z scores indicated that all D statistics were significant, ie Z score greater than 3 or less than -3 (Fig 10). These analyses suggest admixture into the southeastern dingo (Alpine) population from Vietnam and Borneo village dogs but not from European dogs (Fig 10). In contrast, D statistics reveal possible shared genetic ancestry or admixture into northwestern (and Fraser Island) dingoes from Portugal village dogs and Australian cattle dogs (Fig 10).

## Discussion

### Current biogeographic structure in dingoes

There is now strong evidence of at least three genetically distinct dingo lineages in Australia: SE dingoes, FI dingoes, NW dingoes, [62, 67]. NGSDs form a separate but closely related distinct lineage (Figs 9 and 10). Population clustering analyses indicated that the SE and FI dingoes form discrete groups, whilst the dingoes from the Kimberley, Simpson Desert and Gibson Desert cluster together in a NW population (Figs 3–5). Principal components analysis also identified the presence of three dingo population clusters distinguishable from the NGSD (Figs 6 and 7). Interestingly, whilst the NGSD, FI and NW clusters were tightly clustered, the SE samples were not. This suggests that the SE population is more heterogeneous than the other dingo and NGSD populations perhaps due to greater dispersal and mixing in this population [29] or introgression from modern domestic dogs.

Phylogenetic analyses suggest that biogeographical patterns depicted in the genome wide SNP data (Figs 8 and 9) are similar to those observed in mitochondrial and nuclear gene data [62], with two key differences. The presence of at least two dingo populations is consistent across all genetic markers [62, 67]. However, mitochondrial data suggested that SE and FI dingo populations are closely related whilst the whole genome SNP data, presented here, and Y-chromosome data suggest that FI dingoes may be more closely related to the NW dingo populations [62, 67]. Bootstrap support for the FI/NW population grouping is low at 60–73% suggesting there is some uncertainty concerning the relationship of the FI population to the other dingo populations (Figs 8 and 9). This uncertainty could be a reflection of the different evolutionary histories of the maternal and autosomal genetic markers. One hypothesis to explain the results is that the FI population is the product of an initial foundation from the southeastern mitochondrial lineage, followed by paternal introgression from the northwestern lineages. It is possible that historical human movements between mainland Australia and Fraser Island by Indigenous Australians may have facilitated historical paternal introgression. Clustering, phylogenetic, F_ST_ and PCA analyses suggest that the FI dingo population currently forms a discrete lineage with little recent gene flow from the mainland.

### Are dingo populations evolutionarily significant units (ESUs)?

A population is an evolutionarily significant unit (ESU) if it is: (1) geographically separated; (2) is genetically differentiated due to reduced gene flow or; (3) carries locally adapted phenotypic traits [90, 91]. *F*_*ST*_ values between the four population clusters suggest that gene flow between dingo populations is low, and that populations are highly divergent (Table 3). An alternative hypothesis is that populations are genetically intermediate. Published mitochondrial data suggests that there is strong geographic subdivision between dingo lineages and that plausibly the lineages diverged outside Australia [62]. SNP and Y-chromosome data indicates that there may be some admixture between geographically subdivided lineages, specifically between the FI and SE populations and between the NW and SE populations [67]. SNP data also shows some evidence of limited gene flow or ancestry sharing between the SE and FI populations and NW (Simpson Desert) dingoes (Figs 3 and 5). This may be the result shared ancestry, historical mixing between dingo populations or modern gene flow. Higher levels of dispersal in southeastern Australia due to widespread lethal control and the disruption of dingo social structures may also have modified historical gene flow patterns either driving or inhibiting gene flow between dingo populations [29]. Molecular dating suggests the genetic lineages diverged approximately 8,000 years BP, significantly before the presence of modern dispersal barriers such as the ‘dingo fence’ [62]. Indeed, whilst the ‘dingo fence’ fence does restrict the movement of dingoes it is unlikely to be completely impenetrable and so restricted gene flow may occur [7]. Phenotypic differences have also been observed between dingoes in different geographic regions, this is perhaps the result of local adaption or differences in evolutionary history [92]. Ultimately, there is evidence of genetic and possibly phenotypic differentiation between extant dingo populations, this combined with geographic isolation, supports the designation of three dingo ESUs: SE, FI and NW. The maintenance of population structure in the absence of obvious physical barriers suggests that introduction history, behavior and/or biology is shaping and maintaining biogeographic differentiation in the dingo. As distinct ESUs, the three dingo populations should ideally be managed separately.

### Hybridization or shared ancestry

We used Bayesian clustering, PCA and D-statistics to investigate hypotheses concerning the evolutionary history of dingoes and the presence of domestic dog introgression Hybridization between dingoes and dogs is considered to be widespread in southeastern Australia [33]. There are fears that the dingo will lose its identity because of ongoing hybridization with European dogs [33]. At first, we hypothesized that dingoes in southeastern Australia may carry a low level of modern European domestic dog introgression and that this may be responsible for the observed heterogeneity in our genetically ‘pure’ southeastern dingo samples (Fig 6). This was initially consistent with clustering analyses with K = 5, which showed some shared ancestry between southeastern dingoes and Portugal village dogs (Fig 5). However, clustering analyses with K = 7 suggest that southeastern dingoes share ancestry with South East Asian village dogs rather than European breed dogs (Fig 5). Additionally, D statistics provide additional evidence that the southeastern dingoes share ancestry with South East Asian dogs, represented here by village dogs from Borneo and Vietnam (Fig 10). One hypothesis is that there may have been gene flow between island South East Asian dogs and dingoes in the last few hundred years. However, ethnographic and human DNA studies suggest that there is only limited evidence of contact between South East Asia and Australia during this time period [93–98]. The most logical hypothesis is that the southeastern dingoes ancestors originated in South East Asia and thus share ancestry with South East Asian dogs [48, 50, 53, 62, 67]. Interestingly this signal of shared ancestry with South East Asian dogs is observed only in southeastern dingoes (Alpine), suggesting that perhaps the two dingo populations have differing ancestral populations [62, 67].

Clustering analyses did not find evidence of admixture within northwestern or Fraser Island dingoes from Asian village dogs or European domestic dogs (Fig 5). This is consistent with evidence that domestic dog hybridization is less widespread in northwestern and central Australia [33]. However, D statistics identified the presence of ancestry sharing from European derived dogs (Portugal village dogs and Australian cattle dogs) into northwestern and Fraser Island dingoes (Fig 10). Perhaps this ancestry sharing between Australian cattle dogs and dingoes is a result of dingoes being used in the foundation of the breed. Indeed, the lack of admixture signal observed in clustering analyses suggests that any introgression was likely historical. One hypothesis is that northwestern and Fraser Island dingoes share ancestry with a dog population that shares more affinity with European dogs than Asian dogs, for example from India or Java. Or share ancestry with dogs from an un-sampled South East Asian region. Intriguingly this might fit with evidence of a Pama–Nyungan language expansion in Northern Australia during the Holocene period [99] or a possible human immigration from India [100]. However, these hypotheses are controversial [96, 98, 101–103]. Indeed, PCA analyses indicate that all dingoes are more closely related to South East Asian dogs than to European dogs (Fig 7). As first suggested by Cairns and Wilton [63], these differences in ancestry sharing patterns between the distinct dingo populations is likely evidence that the dingo lineages diverged outside of Australia and had different evolutionary origins in Asia. To address this uncertainty future research should aim to incorporate additional dingoes from across Australia and a larger number of dogs from both South East Asia and modern European domestic dog breeds.

### Inbreeding in wild dingo populations

Inbreeding coefficients can be used to assess the genetic health of a population or species [38, 39]. High homozygosity levels may indicate when a population is genetically unhealthy and at risk of inbreeding depression. Inbreeding and resulting inbreeding depression can lead to the decline and/or extinction of the species or population [37–43]. Domestic dogs, specifically purebred domestic breeds, typically have elevated levels of inbreeding due to the effect of human artificial selection, population bottlenecks and line breeding [104]. Inbreeding coefficient values for domestic dogs are reported to range between −0.2 and 0.19 [104–107]. Inbreeding in wild canid populations is reported to be lower than domestic dogs, with coyotes *F*_*IS*_ = 0.04 [105] and wolves *F*_*IS*_ = 0.06–0.08 [105, 108, 109]. An inbred Scandinavian wolf population provides an example of how inbreeding depression can lead to increasing physiological anomalies including lower lifespan and elevated incidences of congenital defects [110]. The SE and Simpson Desert (NW) dingo populations have average inbreeding coefficient values similar to those of wild canid populations, whilst the Kimberley (NW) and Gibson Desert (NW) populations have slightly elevated average inbreeding levels (Table 2). Simpson Desert (NW) and SE dingo populations are under higher levels of human-mediated disruption, with ongoing management action plans possibly contributing to elevated dispersal/immigration events in these populations [29].

We observed high inbreeding levels in Fraser Island dingoes, *F*_*IS*_ = 0.647–0.732 (Table 2). Comparison of the Fraser Island sample locations to reported dingo pack territory boundaries suggests that these dingoes were from different natal territories and thus are unlikely to be siblings (Fig 2) [89]. High inbreeding suggests that the FI dingo population may not be genetically healthy and the viability of the population may be compromised. This high inbreeding could be the result of a low foundation population followed by a long period of isolation and may have been exacerbated by lethal control activities. The *F*_*IS*_ range observed in the NGSD, *F*_*IS*_ = 0.457–0.671, was also extremely high. NGSD in captive populations were likely experiencing high inbreeding due to a limited founder population [111]. However, sample sizes were quite small. It is also possible that inbreeding coefficient levels were raised due to the ascertainment bias caused by phylogenetic distance between dingoes and the dog breeds used to develop the Illumina HD Canine Chip. SNP array technologies have been successfully applied to other canids such as wolves and coyotes, with limited ascertainment bias observed [54, 112, 113]. Further research and sampling is needed to examine these patterns of inbreeding.

### Conservation management implications

As the three dingo populations, SE, FI and NW, may be considered separate ESUs, existing management and conservation strategies may need to be revised. Dingoes are widespread across the mainland (Fig 1); however, they are subject to high levels of lethal control particularly in eastern Australia [7, 92]. Dingoes in southeastern Australia are also under increasing pressure hybridization with domestic dogs [7, 33]. Recently, calls have been made for revised management strategies to protect the unique identity of southeastern dingoes [67]. This may include measures such as reducing lethal management around national parks, encouraging landholders to uptake predator friendly farming practices and neutering pet dogs to reduce future hybridization. Government agencies should also undertake surveys to identify high genetic integrity dingo populations and lethal control management may need to be reevaluated in regions with high genetic integrity dingo populations.

Hybridization with domestic dogs is considered a serious issue for the conservation of dingoes [32, 33]. Comparison of dingoes to domestic dogs using whole genome SNP data may help uncover markers for distinguishing dingoes from their hybrids. To investigate SNP markers useful in distinguishing dingoes, hybrids and domestic dogs further research incorporating dingoes, known hybrids, South East Asian village dogs and Australian domestic dogs will be necessary. This may lead to improvements in the accuracy of genetic testing for hybridization and also knowledge concerning the prevalence and factors affecting hybridization between dingoes and domestic dogs. New frameworks for the conservation and management of dingoes considering hybridization may need to be developed.

This study has important implications for the management of dingoes on Fraser Island. Current population estimates for dingoes on Fraser Island are approximately 100–200 individuals [45, 89, 114], down from approximately 300 in the 1990s [115]. The FI dingo population is thought to serve as an important reservoir of pure dingoes isolated from mainland populations and relatively free from hybridization [32, 46]. High levels of inbreeding, as observed by this study (Table 2), are concerning for the persistence and sustainability of dingoes on the Island. The Fraser Island Dingo Management Plan reports that the primary aim of the strategy is to “ensure conservation of a sustainable dingo population” [116]. Allen et al. [89] argue that the Fraser Island dingo population is demographically stable and thus sustainable. Our data indicate that the population may be compromised by severe inbreeding and could require genetic rescue. Without further information regarding the genetic health of the population, management decisions to cull dingoes should be made cautiously. This data highlights the need for regular genetic monitoring on Fraser Island to track genetic diversity and possible inbreeding depression.

Genetic rescue has been used to safeguard inbred populations of threatened species around the world, including Florida panthers (*Puma concolor coryi*), mountain pygmy possums (*Burramys parvus*) and Rocky Mountain bighorn sheep (*Ovis canadensis*) [44, 117–120]. This controversial tool may need to be considered during the ongoing management of both Fraser Island dingoes and NGSD. Genetic rescue of the Fraser Island dingo population will need to carefully consider the most suitable migrant population given that Fraser Island dingoes may be considered an ESU distinct from both mainland dingo lineages. Introduction of new genetic lineages to the captive NGSD population may also be required to avoid inbreeding depression; however, this will likely prove difficult given sightings of wild NGSD have been scarce since the 1950s and due to the remote and rugged mountain habitat [111].

## Conclusions

Whole genome SNP data indicate that there are at least three divergent dingo populations forming discrete conservation units (ESUs). This builds upon previously published mitochondrial and Y-chromosome studies [62, 67]. FI dingoes form a distinct population from the mainland dingo lineages. However, historical human movements between mainland Australia and Fraser Island may have facilitated immigration between mainland and FI populations. There is some evidence of ancestry sharing between southeastern dingoes and South East Asian village dogs, either due to ancestral origin or historical admixture. We also identified some evidence of admixture into northwestern and Fraser Island dingoes from European derived dogs. This might be the result of geographical origin, historical admixture or the use of dingoes in the foundation of some modern dog breeds. The different admixture patterns indicate that the three dingo populations may have different evolutionary histories or origins.

This study highlights the need to conserve dingoes from all geographical regions of Australia to preserve the full range of genetic diversity and identity of the dingo. However, dingoes in southeastern Australia are in urgent need of protection due to the increased pressures of lethal control and hybridization in the region. Concerningly, FI dingo populations and the captive NGSD population appear to be under threat of inbreeding. Management strategies may need to be adapted to preserve and improve the genetic health of these important canid populations.

## Supporting information

S1 Fig Cross validation error for ‘Dataset A’ ADMIXTURE analyses. Cross validation errors for each K value were averaged across ten independent runs in ADMIXTURE v1.23. Error bars represent standard error calculated across the ten runs. Cross validation error was lowest for K = 4.
(PDF)

S2 Fig Cross validation error for ‘Dataset C’ ADMIXTURE analyses. Cross validation errors for each K value were averaged across ten independent runs in ADMIXTURE v1.23. Error bars represent standard error calculated across the ten runs. Cross validation error was lowest for K = 5.
(PDF)

S1 Table Sample data. Identifier, Geographical Locale, Latitude, Longitude and Genetic Identity for genotyped samples.
(PDF)
