# Supplementary figures and images for: Elucidating biogeographical patterns in Australian native canids using genome wide SNPs
Source: PLoS One. 2018 Jun 11;13(6):e0198754. doi: 10.1371/journal.pone.0198754 (PMC5995383; doi:10.1371/journal.pone.0198754)

Cross Validation Error

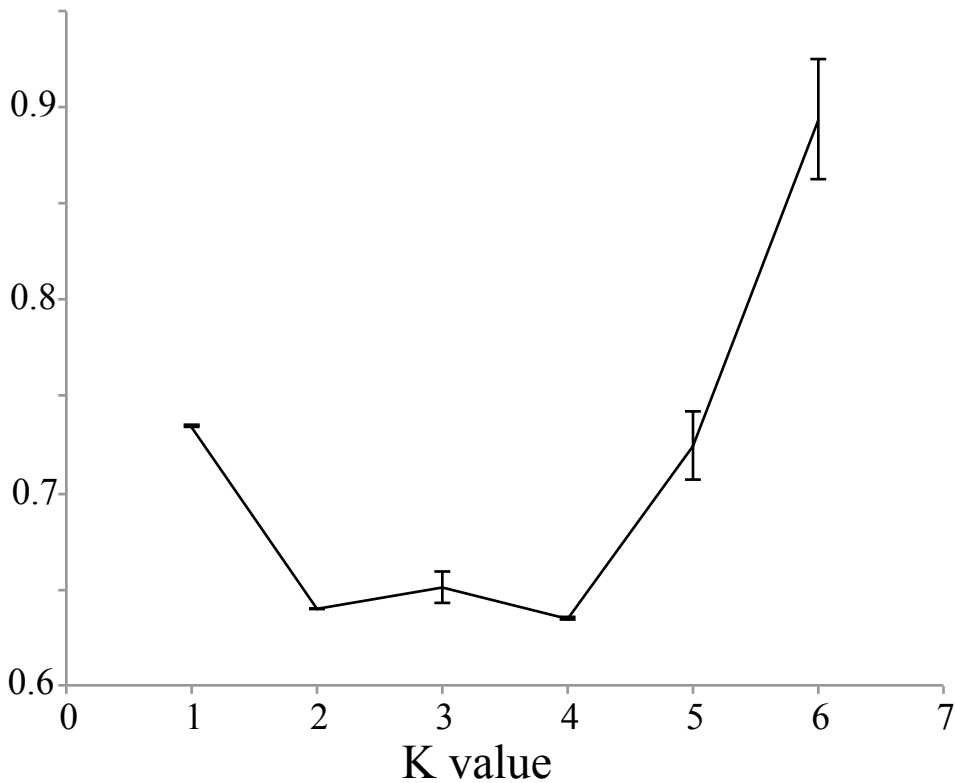

Supplement: S1 Fig — Cross validation errors for each K value were averaged across ten independent runs in ADMIXTURE v1.23. Error bars represent standard error calculated across the ten runs. Cross validation error was lowest for K = 4. (PDF) [file pone.0198754.s001.pdf]

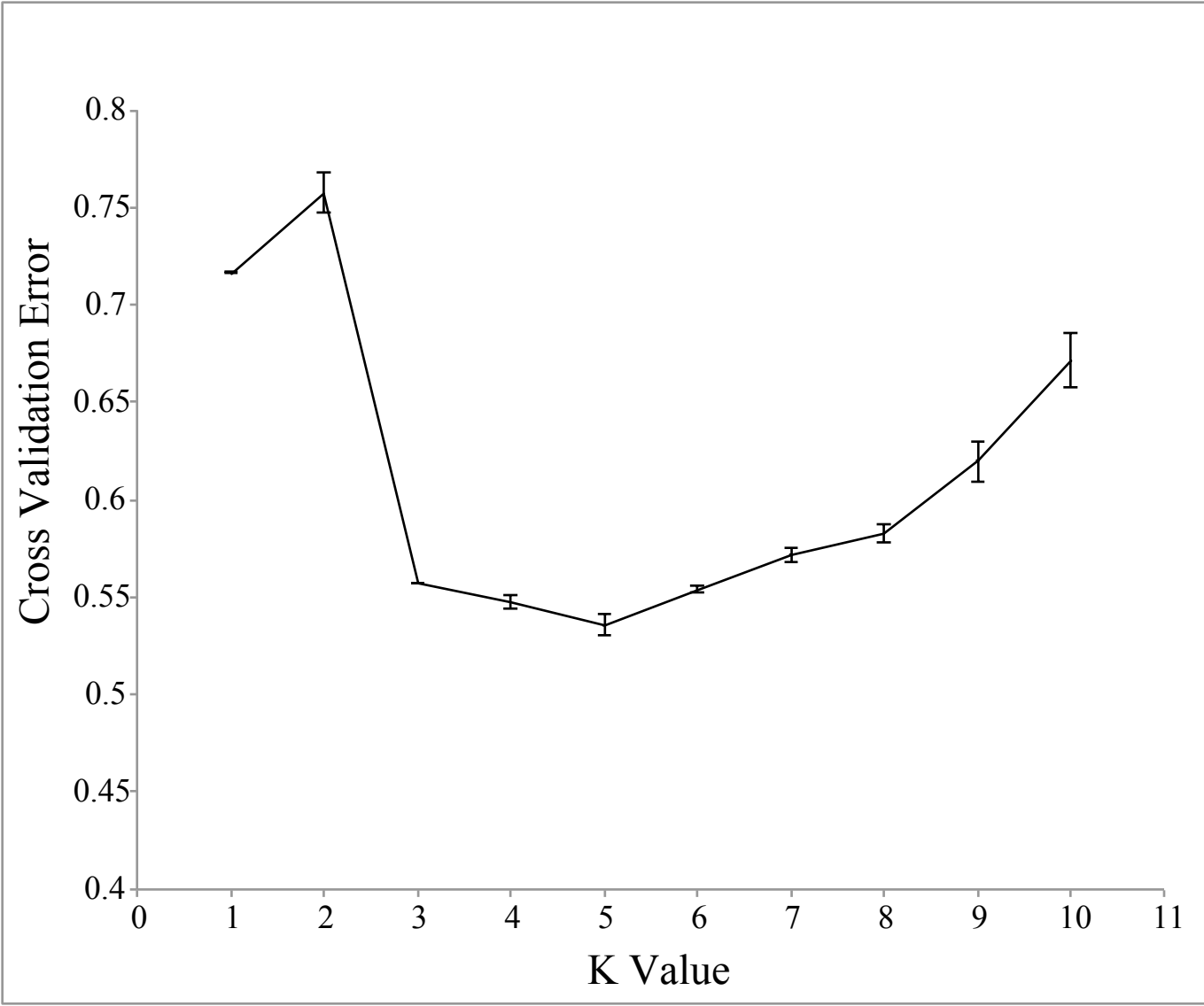

Supplement: S2 Fig — Cross validation errors for each K value were averaged across ten independent runs in ADMIXTURE v1.23. Error bars represent standard error calculated across the ten runs. Cross validation error was lowest for K = 5. (PDF) [file pone.0198754.s002.pdf]
