# Supplementary material for: Elucidating biogeographical patterns in Australian native canids using genome wide SNPs
Source: PLoS One. 2018 Jun 11;13(6):e0198754. doi: 10.1371/journal.pone.0198754 (PMC5995383; doi:10.1371/journal.pone.0198754)
Supplement: S1 Table — Identifier, Geographical Locale, Latitude, Longitude and Genetic Identity for genotyped samples. (PDF) [file pone.0198754.s003.pdf]

**S1 Appendix** Sample data; Identifier, Geographical Locale, Latitude, Longitude and Genetic Identity

| Name | ID | Canid | Sex | State | Longitude | Latitude | CR haplotype (collapsed) | CR haplotype (gaps) | MtDNA lineage | MtDNA type | Y-Chr Haplotype | SNP cluster |
|---|---|---|---|---|---|---|---|---|---|---|---|---|
| Alpine 1 | 96.2 | dingo | M | VIC | -37.29 | 148.33 | A209 | A209 | SE | a9 | H60-k11 | SE |
| Alpine 2 | WD170 | dingo | M | NSW | -36.46 | 148.26 | A29 | A29 | SE | a3 | H1-k1 | SE |
| Alpine 3 | 44.5 | dingo | M | ACT | -35.84 | 148.98 | A29 | A29 | SE | a3 | H1-6t | SE |
| Alpine 4 | 135.10 | dingo | F | VIC | -37.07 | 148.58 | A29 | A179 | SE | a3 | | SE |
| Alpine 5 | 119.1 | dingo | M | VIC | -36.17 | 147.99 | A29 | A179 | NW | d23 | H60-k11 | SE |
| Fraser 3 | 184.4 | dingo | M | QLD | -25.18 | 153.28 | A29 | A179 | SE | f1 | | FI |
| Fraser 4 | 184.1 | dingo | M | QLD | -25.70 | 153.03 | A29 | A179 | SE | f1 | H60-n25 | FI |
| Fraser 5 | 21.4 | dingo | F | QLD | -25.79 | 153.08 | A29 | A179 | SE | f1 | | FI |
| Fraser 7 | 184.2 | dingo | M | QLD | -25.6 | 153.09 | - | - | - | - | - | FI |
| Gibson 1 | 9.40 | dingo | F | WA | -26.22 | 121.55 | A203 | A203 | NW | d5 | | NW |
| Gibson 2 | 19.84 | dingo | F | WA | -27.30 | 123.06 | A29 | A29 | NW | d5 | | NW |
| Gibson 3 | DE17 | dingo | M | WA | -26.42 | 121.67 | A200 | A200 | NW | d5 | H60-n24 | NW |
| Gibson 4 | DE13 | dingo | F | WA | -25.45 | 122.90 | A29 | A29 | NW | d5 | | NW |
| Gibson 5 | 18.35 | dingo | F | WA | -25.08 | 122.05 | A200 | A200 | NW | d5 | | NW |
| Kimberley 1 | 3.45 | dingo | F | WA | -15.10 | 125.53 | A29 | A29 | NW | d5 | | NW |
| Kimberley 2 | 7.27 | dingo | M | WA | -16.63 | 124.88 | A9 | A9 | NW | d7 | H3-n4 | NW |
| Kimberley 3 | 19.11 | dingo | M | WA | -17.47 | 125.08 | A9 | A9 | NW | d7 | H3-n20 | NW |
| Kimberley 4 | 4.55 | dingo | F | WA | -15.35 | 126.10 | din27 | din27 | NW | d5 | | NW |
| Simpson 1 | 182.6 | dingo | F | SA | -26.65 | 140.35 | A29 | A179 | NW | d5 | | NW |
| Simpson 2 | X1777 | dingo | M | NT | 25.35 | 133.71 | A200 | din35 | NW | d5 | H60-9k | NW |
| Simpson 3 | 142.3 | dingo | F | SA | -27.94 | 134.74 | A200 | A200 | NW | d4 | | |
| Simpson 5 | X1783 | dingo | F | NT | -24.23 | 131.42 | A200 | A200 | NW | d5 | | NW |
| Northwestern 2 | 11.55 | dingo | M | WA | -27.41 | 122.36 | A29 | A29 | NW | d15 | H60-n22 | NW |

**S1 Appendix** Sample data; Identifier, Geographical Locale, Latitude, Longitude and Genetic Identity

| Name | ID | Canid | Sex | State | Longitude | Latitude | CR haplotype (collapsed) | CR haplotype (gaps) | MtDNA lineage | MtDNA type | Y-Chr Haplotype | SNP cluster |
|---|---|---|---|---|---|---|---|---|---|---|---|---|
| Northwestern 9 | 24.94 | dingo | F | WA | -17.27 | 122.57 | A29 | A29 | NW | d15 | | NW |
| NGSD A | Subu | NGSD | M | PNG | - | - | - | - | - | - | - | NGSD |
| NGSD B | Crosby | NGSD | M | PNG | - | - | - | - | - | - | - | NGSD |
| NGSD C | Keba | NGSD | M | PNG | - | - | - | - | - | - | - | NGSD |
| NGSD D | Hali | NGSD | F | PNG | - | - | - | - | - | - | - | NGSD |
| NGSD E | Roux | NGSD | M | PNG | - | - | - | - | - | - | - | NGSD |
